# Socio-demographic factors and healthy lifestyle behaviours among Malaysian adults: National Health and Morbidity Survey 2019

**DOI:** 10.1038/s41598-022-20511-1

**Published:** 2022-10-04

**Authors:** Wan-Fei Khaw, Nur Hamizah Nasaruddin, Nazirah Alias, Yee Mang Chan, LeeAnn Tan, Siew Man Cheong, Shubash Shander Ganapathy, Muhammad Fadhli Mohd Yusoff, Heng Yaw Yong

**Affiliations:** 1 Institute for Public Health, National Institutes of Health, Ministry of Health Malaysia, Shah Alam, Selangor Malaysia; 2 Bentong Health Clinic, Bentong District Health Office, Ministy of Health Malaysia, Bentong, Pahang Malaysia; 3 Division of Nutrition and Dietetics, School of Health Sciences, International Medical University, Kuala Lumpur, Malaysia

**Keywords:** Health care, Risk factors

## Abstract

This study aimed to investigate the association between socio-demographic factors and designated healthy lifestyle behaviours in a nationally-representative sample of Malaysian adults aged 18 years and above. Secondary data involving 7388 participants aged 18–96 years from the National Health and Morbidity Survey 2019, a national cross-sectional survey, was used in this study. A healthy lifestyle score (0–5 points) was calculated based on five modifiable lifestyle factors: non-smoker, body mass index < 25 kg/m^2^, physically active, moderate (or less) alcohol intake, and daily consumption of ≥ 5 servings of fruits and vegetables. Associations between socio-demographic factors and healthy lifestyle behaviours were examined using multinomial logistic regression adjusted for sampling design. About 30.6% of the participants met at least four out of the five healthy lifestyle factors. In multinomial model, subjects who were female (aOR = 3.26, 95%CI = 2.58, 4.12), of Chinese (aOR = 2.31, 95%CI = 1.62, 3.30 or other ethnicity (aOR = 1.44, 95%CI = 1.05, 1.98), and aged 18–30 years (aOR = 1.74, 95% CI = 1.12, 2.71) showed significant association with achieving healthy lifestyle compared to male, Malay and ≥ 61 years old as reference categories. Our results indicated that gender, age and ethnicity associated with healthy lifestyle behaviours. Information on the influence of socio-demographic factors on the prevalence of healthy lifestyles will facilitate the development of effective intervention strategies to improve the adaptation of healthy lifestyle practices.

## Introduction

Healthy lifestyle habits are important for preventing disease, improving health, and maintenance of overall well-being throughout one’s life^1^. In general, healthy lifestyle habits include adopting a healthy diet, abstaining from smoking, maintaining a healthy weight, avoiding harmful alcohol consumption, and being physically active. Leading a healthy lifestyle also reduces one’s risk of developing major chronic diseases. While the aforementioned modifiable lifestyle factors are individually associated with a decreased risk of major chronic diseases and a longer life expectancy, several studies have found that combined healthy lifestyle habits have a greater impact on increased life expectancy than on their own^2^. Other studies have also found that a combination of healthy lifestyle factors is associated with improved survival^3^ and reduced risk of major chronic diseases, such as coronary heart disease, stroke, heart failure and diabetes^4–6^. Adherence to a combination of five healthy lifestyle factors reduced mortality risk by 61%^3^. In previous studies among adults in China^7,8^ and cancer survivors^9^, researchers looked into the effects of combined healthy lifestyle factors in lowering overall and cause-specific mortality. Using measures of healthy lifestyle, including exercise, diet, sleep, alcohol consumption, and smoking, it found that 7.7% of adults reported practicing five healthy behaviours^10^. In another study using European Social Survey data observed that around 5.8% of European adults achieving all five healthy behaviours. Finland with 9.2% and United Kingdom with 8.6% were those the highest prevalence while Hungary with 1.3% and Czech Republic with 1.9% were the lowest prevalence of five healthy behaviours^11^. To date, there have been no studies conducted in Malaysia focusing on the effects of combined healthy lifestyle factors. There is also a lack of information on the prevalence of healthy lifestyle habits or practices among Malaysian adults.

Numerous studies have established a link between the prevalence of adopting healthy lifestyle habits and socio-demographic factors. In Germany, it was found that healthy lifestyle habits differ according to socio-demographic characteristics such as age, sex and educational levels^12^. Other studies found that higher socioeconomic status was associated with combinations of healthy lifestyle habits, decreased risk of cardiovascular disease, and lower mortality rates^13^. Information on the influence of socio-demographic factors on the likelihood of leading a healthy lifestyle amongst the local population is important for public health policy development, as it identifies relevant target areas for appropriate individual- and population-level interventions.

In Malaysia, there are no nationally-representative studies on the prevalence of healthy lifestyle behaviours and its socio-demographic correlates in adult populations. The purpose of this study, therefore, was to assess the prevalence of healthy lifestyle behaviours based on data from the National Health and Morbidity Survey (NHMS)—a nationwide health study in Malaysia—and to identify socio-demographic factors associated with leading a healthy lifestyle in a nationally-representative sample of Malaysian adults.

## Methods

### Data and participants

This study utilized secondary data from NHMS 2019. The NHMS is a national survey coordinated by the Institute of Public Health, Ministry of Health Malaysia, and has been conducted annually since 2011. This study is a cross-sectional survey with the aims of determining Malaysians’ health status, monitoring disease trends, and assessing healthcare utilization in Malaysia. The NHMS 2019 employs a nationally representative sample of the non-institutionalised general population of Malaysians of all ages in Malaysia. This study employs two-stage stratified random sampling to ensure the resulting sample was representative of the country. The country’s population is stratified by urban and rural regions in each of the 16 states or federal territories, hence a total of 29 strata. The first stage involves systematic random sampling of Enumeration Blocks (EB) within each stratum. The second stage involves random sampling of twelve living quarters (LQ) within each selected EB. For the entire country of Malaysia, a total of 475 Enumeration Blocks (EB) were selected proportionate to the population size of each stratum. A total of 5676 LQs were selected from the selected EBs. Further details of the NHMS 2019 study have been described in the NHMS 2019 technical report^14^. Between July and October 2019, 14,965 participants were recruited (response rate of 87.2%). All participants signed an informed consent form.

For the present study, participants under the age of 18 years were excluded (n = 4493), as the study’s focus was on the adult population. Respondents with missing socio-demographic data and healthy lifestyle data were excluded (n = 3084). After these exclusions, 7388 respondents were included in the present analyses.

### Assessment of lifestyle factors and socio-demographic variables

Body weight, height and body mass index (BMI) were determined. Body weight was measured in kilograms using a digital weighing machine (TANITA HD-319) and the height in centimetres was measured using SECA Stadiometer 213. The BMI was computed by dividing weight in kilograms by the squared height in metres. BMI was then classified according to the WHO BMI guidelines: (< 25 kg/m^2^ as underweight to normal weight, ≥ 25 kg/m^2^ as overweight or obese)^15^.

Four questions were used to assess fruit and vegetable consumption: (1) “In a typical week, how many days did you consume fruits?” (2) “Usually on the day you eat fruits, how many servings of fruits did you eat in a day?” (3) “In a typical week, how many days did you eat vegetables?” (4) “Usually on the day you eat vegetable, how many servings of vegetables did you eat in a day?” Food photographs were used to assist respondents in recalling the serving size of fruits and vegetables consumed. The photographs depicted a single serving of commonly consumed fruits and vegetables, including one slice of watermelon, one medium-sized orange, one cup of chopped raw leafy green vegetables and a half cup of other vegetables, cooked or chopped raw such as carrot. The total number of servings of fruits and vegetables consumed per day was calculated using responses to these four questions. Data from the first two questions were used to estimate respondents’ fruits intake. Another two questions were used to estimate respondents’ vegetable intake. The number of days in a week was multiplied by number of servings to estimate the number of servings per week that respondents consumed. Responses for the past 7-day questions were then divided by 7 to determine daily intake. The number of servings/day for fruits and vegetable intake were summed to estimate the servings of fruits and vegetables intake. The range of score was 0 to 12 servings/day. Based on the Malaysian Dietary Guidelines, consumption of ≥ 5 servings of fruits and vegetables per day was defined as adequate while consumption of < 5 servings of fruits and vegetables per day was defined as inadequate^16^.

The validated short version of the International Physical Activity Questionnaire (IPAQ) was used to assess levels of physical activity^17^. The IPAQ short form was developed to estimate overall physical activity levels through the assessment of three specific types of physical activity (low, moderate and vigorous-intensity activities) across a broad range of domains (home, at work, sitting, moderate leisure, vigorous leisure, transportation and other activities). Physical activity was quantified in terms of metabolic equivalents (MET minutes, or METs)^18^. The minutes and days of a week spent on low, moderate, and vigorous intensity activities were multiplied by 3.3, 4.0 and 8.0 respectively, to compute MET scores for each activity. The total physical activity score was calculated as the sum of all MET scores from three sub-components. MET score ranges from 0 to 25,704. Participants in this survey were classified as “active” if they had a total physical activity score of at least 600 METs per week according to the WHO’s recommendation^19^. Those who did not meet the weekly requirement of 600 METs were classified as “inactive”.

The Malay version of the Alcohol Use Disorder Identification Test (AUDIT-M) questionnaire was used to assess alcohol consumption^20^. The AUDIT is a validated 10-item questionnaire that includes alcohol consumption, drinking behaviours, and alcohol-related problems. Scores were classified as low risk (score ranging from 1 to 7) or risky (score ranging from 8 to 40). Non-drinker was defined as having had no alcohol consumption for the past 12 months, which includes never drinkers.

The smoking status was divided into two categories: smoker (those who smoked tobacco products on a daily or occasional basis at the time of the survey) and non-smoker (those are not currently smoked any tobacco products).

### Construction of a healthy lifestyle score

BMI, diet, physical activity, cigarette smoking, and alcohol consumption were all used to calculate a healthy lifestyle score^12^. The composite score’s five modifiable lifestyle factors were chosen based on national recommendations for non-communicable disease risk factors^21^ and epidemiological evidence in Asians^22,23^. For each of these components, a binary variable was created with one point assigned (0 otherwise) if the participant meets each criteria as follows: a BMI of < 25 kg/m^2^, performs regular physical activity, has moderate (or less) alcohol consumption, consumes at least 5 servings of fruits and vegetables, and does not smoke^12^. The healthy lifestyle score was calculated as the sum of the binary point system, ranging from 0 to 5 points. Participants were classified into three groups: unhealthy, moderately healthy and healthy. Individuals with healthy lifestyle scores of 0–2 were classified as ‘unhealthy’, those with a healthy lifestyle score of 3 were classified as ‘moderately healthy’, and those with healthy lifestyle scores of 4–5 were classified as the ‘healthy’ group.

### Socio-demographic variables

The independent variables, which were socio-demographic variables, were used as categorical variables in the analyses. Age was captured in the data as a continuous variable and recoded into three categories (18–30 years, 31–60 years, and 60 years and above). Highest educational levels were combined into three categories; primary, secondary and tertiary. ethnicity (Malay, Chinese, Indian and others), educational level (no formal education/ primary, secondary and tertiary), residential area (urban and rural) and marital status (single, married and divorced/widowed). Income information defined as monthly household income, was collected. The income categories were divided into three categories; bottom 40% (B40), middle 40% (M40), and top 20% (T20) of household income.

### Statistical analyses

Data were analysed using IBM SPSS version 22; p-values of < 0.05 were considered statistically significant. Weighting was applied to take into account the complex study design.

A weighting factor was applied to each respondent to adjust for non-response and for the varying probabilities of selection. The weight used for estimation was given by:

$$ {\text{W }} = {\text{ W1 }} \times {\text{ W2 }} \times {\text{ F }} \times {\text{ PS}} $$

W1 = the inverse of probability of selecting the EBs, W2 = the inverse of probability of selecting the LQs within the EBs, F = the inverse of an EBs, LQs and individual level non-response adjustment factor, PS = a post stratification adjustment factor calculated by strata and gender.

Survey-weighted descriptive statistics were computed to provide nationally representative estimates. Survey-weighted descriptive analyses of socio-demographic factors related with healthy lifestyle behaviours (unhealthy, moderate healthy and healthy) were done. For categorical variables, frequency and weighted percentage were displayed. Rao-Scott Chi-square test was conducted to identify bivariate associations between socio-demographic factors and healthy lifestyle groups. Multinomial logistic regression adjusted for sampling design was used to determine the association of socio-demographic factors with the moderately healthy and healthy lifestyle behaviours, using the unhealthy lifestyle group as the reference group. All predictors were included in the baseline-category logic model to estimate the adjusted odd ratio (aOR) and 95% confidence interval (CI).

### Ethics approval and consent to participate

All participants signed an informed consent form. The NHMS 2019 was approved by the Medical Research and Ethics Committee of Ministry of Health Malaysia (NMRR-18-3085-44207). When conducted the analysis for this study in 2022, informed consent of the study participants was not required because the database used in this study consists of de-identified secondary data released for research purposes. This study was carried out in accordance with the ethical standards of the Declaration of Helsinki and Ministry of Health Malaysia guidelines and regulations.

## Results

Table 1 presents the general characteristics of study participants. A total of 7388 participants were included in this study. Most of them (54.3%) were in the 31–60 years age group. More than half (50.4%) of them were female. Most participants (70.7%) had at least secondary-level education. Participants were 56.3% Malay, 18.4% Chinese, 5.7% Indians and 19.6% of other ethnic groups, which includes indigenous Sabah and Sarawak people, as well as aborigines. Furthermore, most participants were married (65.4%), urban residents (78.9%) and fell in the bottom 40% household income group (64.4%).

**Table 1:** Socio-demographic characteristics of the study population (n = 7388).

Variables	Count (n)	Weighted percentage, % (95% CI)
**Age group (years)**		
18–30	1650	33.7 (31.5–35.9)
31–60	4334	54.3 (52.2–56.4)
≥ 61	1404	12.0 (10.9–13.2)
**Gender**		
Male	3312	49.6 (47.8–51.3)
Female	4076	50.4 (48.7–52.2)
**Ethnicity**		
Malay	5052	56.3 (52.3–60.1)
Chinese	774	18.4 (15.0–22.2)
Indian	454	5.7 (4.5–7.4)
Others	1108	19.6 (17.2–22.3)
**Marital status**		
Single	1504	28.3 (26.3–30.3)
Married	5194	65.4 (63.3–67.4)
Divorced/widowed	690	6.3 (5.6–7.1)
**Education level**		
No formal education/primary	1904	21.6 (19.7–23.6)
Secondary	3573	49.1 (47.1–51.2)
Tertiary	1911	21.6 (19.7–23.6)
**Residential area**		
Urban	4549	78.9 (77.4–80.3)
Rural	2839	21.1 (19.7–22.6)
**Household income**		
Bottom 40%	4957	64.4 (61.3–67.3)
Middle 40%	1804	26.1 (23.7–28.6)
Top 20%	627	9.5 (7.8–11.6)

Participants were predominantly non-smokers (78.8%), non-drinker or normal drinker (98.2%), physically active (78.7%), consumed < 5 servings of fruits and vegetables per day (95.3%) and overweight or obese (50.5%). The prevalence of participants with 0/1, 2, 3, 4, and 5 healthy lifestyle scores were 2.1%, 18.1%, 49.2%, 29.0%, and 1.6%, respectively. Based on the classification used in the present study, 20.2% were in the unhealthy lifestyle group, 49.2% were in the moderately healthy lifestyle group, and another 30.6% were in the healthy lifestyle group (Table 2).

**Table 2:** Healthy lifestyle factors of study population (n = 7388).

Variables	Count (n)	Weighted percentage, % (95% CI)
**Body mass index**		
< 25 kg/m^2^	3259	49.5 (47.4–51.7)
≥ 25 kg/m^2^	4129	50.5 (48.3–52.6)
**Physical activity**		
Active	5760	78.7 (77.0–80.3)
Inactive	1628	21.3 (19.7–23.0)
**Consumption of vegetables and fruits**		
< 5 servings/days	7090	95.3 (94.4–96.0)
≥ 5 servings/days	298	4.7 (4.0–5.6)
**Smoking status**		
Smoking	1380	21.2 (19.5–23.0)
Non-smoking	6008	78.8 (77.0–80.5)
**Alcohol drinking**		
Risky drinker	80	1.8 (1.2–2.5)
Non-drinker/normal drinker	7308	98.2 (97.5–98.8)
**Healthy lifestyle scores**		
0	3	0.1 (0–0.3)
1	133	2.0 (1.5–2.5)
2	1386	18.1 (16.9–19.5)
3	3823	49.2 (47.3–51.1)
4	1956	29.0 (27.1–30.9)
5	87	1.6 (1.2–2.3)
**Healthy lifestyle behaviours**		
Unhealthy (0–2)	1522	20.2 (18.8–21.6)
Moderate healthy (3)	3823	49.2 (47.3–51.1)
Healthy (4–5)	2043	30.6 (28.6–32.7)

Table 3 shows socio-demographic factors across different categories of healthy lifestyle behaviours. Age, gender, ethnicity and marital status were significantly different by healthy lifestyle groups. More participants were female in the moderately healthy and healthy groups than in the unhealthy group. In addition, participants in the unhealthy group were more likely to be 31–60 years of age, male, Malay, and married than those in the healthy group. No other significant differences were found between categories of healthy lifestyle behaviours in terms of educational level, residential area and household income.

**Table 3:** Socio-demographic factors according to healthy lifestyle behaviours.

Variables	Unhealthy, n(%)	Moderate healthy, n(%)	Healthy, n(%)	P value
**Age group (years)**				** < 0.001**
18–30	312 (20.5)	762 (19.9)	576 (28.2)	
31–60	931 (61.2)	2346 (61.4)	1057 (51.7)	
≥ 61	279 (18.3)	715 (18.7)	410 (20.1)	
**Gender**				** < 0.001**
Male	991 (65.1)	1586 (41.5)	735 (36.0)	
Female	531 (34.9)	2237 (58.5)	1308 (64.0)	
**Ethnicity**				** < 0.001**
Malay	1102 (72.4)	2660 (69.6)	1290 (63.1)	
Chinese	128 (8.4)	343 (9.0)	303 (14.8)	
Indian	84 (5.5)	267 (7.0)	103 (5.0)	
Others	208 (13.7)	553(14.5)	347 (17.0)	
**Marital status**				**0.008**
Single	316 (20.8)	704 (18.4)	484 (23.7)	
Married	1083 (71.2)	2763 (72.3)	1348 (66.0)	
Divorced/widowed	123 (8.1)	356 (9.3)	211 (10.3)	
**Education level**				0.079
No formal education/primary	388 (25.5)	1007 (26.3)	509 (24.9)	
Secondary	754 (49.5)	1893 (49.5)	926 (45.3)	
Tertiary	380 (25.0)	923 (24.1)	608 (29.8)	
**Residential area**				0.098
Urban	980 (64.4)	2271 (59.4)	1298 (63.5)	
Rural	542 (35.6)	1552 (40.6)	745 (36.5)	
**Household income**				0.559
Bottom 40%	1006 (66.1)	2605 (68.1)	1346 (65.9)	
Middle 40%	383 (25.2)	910 (23.8)	511 (25.0)	
Top 20%	133 (8.7)	308 (8.1)	186 (9.1)	

Significant values are in bold.

Table 4 shows socio-demographic factors affecting healthy lifestyle behaviours using multinomial logistic regression analysis. The unhealthy lifestyle group was considered as the reference category in the multinomial logistic regression model. In the final model, gender, ethnicity and residential area appeared to be significant predictors of leading a moderately healthy lifestyle (p < 0.05). Women were 2.77 times more likely to achieve a moderately healthy lifestyle (95%CI = 2.28, 3.36). Indian participants were more likely to have moderately healthy lifestyles compared to Malays as reference category (aOR = 1.82, 95%CI = 1.32, 2.52). Participants residing in rural areas were more likely to have moderately healthy lifestyle compared with participants residing in urban areas as reference category (aOR = 1.24, 95%CI = 1.02,1.51).

**Table 4:** Socio-demographic factors associated with healthy lifestyle behaviours categories: multinomial regression analysis.

Variables	Moderate healthy^a^		Healthy^a^	
	Adjusted odds ratio (95% CI)	P value	Adjusted odds ratio (95% CI)	P value
**Age group (years)**				
18–30	1.02 (0.71–1.47)	0.911	**1.74 (1.12–2.71)**	**0.014**
31–60	0.87 (0.68–1.12)	0.284	0.82 (0.61–1.10)	0.183
≥ 61	1.00		1.00	
**Gender**				
Female	**2.77 (2.28–3.36)**	**< 0.001**	**3.26 (2.58–4.12)**	**< 0.001**
Male	1.00		1.00	
**Ethnicity**				
Chinese	1.19 (0.86–1.66)	0.297	**2.31 (1.62–3.30)**	**< 0.001**
Indian	**1.82 (1.32–2.52)**	**< 0.001**	1.49 (0.98–2.27)	0.062
Others	1.13 (0.87–1.48)	0.365	**1.44 (1.05–1.98)**	**0.026**
Malay	1.00		1.00	
**Marital status**				
Single	1.09 (0.71–1.68)	0.690	0.85 (0.51–1.42)	0.539
Married	1.25 (0.92–1.70)	0.149	1.03 (0.73–1.43)	0.887
Divorced/widowed	1.00		1.00	
**Education level**				
No formal education/primary	0.91 (0.69–1.19)	0.472	0.83 (0.58–1.18)	0.296
Secondary	1.06 (0.85–1.31)	0.613	0.87 (0.66–1.16)	0.340
Tertiary	1.00		1.00	
**Residential area**				
Rural	**1.24 (1.02–1.51)**	**0.032**	1.15 (0.89–1.48)	0.286
Urban	1.00		1.00	
**Household income**				
Bottom 40%	1.09 (0.77–1.52)	0.664	0.95 (0.61–1.49)	0.832
Middle 40%	0.97 (0.66–1.41)	0.857	0.82 (0.51–1.34)	0.432
Top 20%	1.00		1.00	

Significant values are in bold.

^a^Reference category = unhealthy.

The multinomial logistic regression analysis found that statistically significant factors associated with leading a healthy lifestyle were age, ethnicity and gender. Participants aged 18–30 years old were more likely to have healthy lifestyle scores of 4 and 5 (aOR = 1.74, 95% CI = 1.12, 2.71) compared to older people ≥ 61 years old as reference category. Women were 3.26 times more likely to achieve healthy lifestyles compared to males as reference category (aOR = 3.26, 95%CI = 2.58, 4.12). Chinese participants (aOR = 2.31, 95%CI = 1.62, 3.30) and participants with other ethnicities (aOR = 1.44, 95%CI = 1.05, 1.98) were more likely to achieve healthy lifestyles compared to Malay participants.

## Discussion

In this cross-sectional study among a nationally representative sample of Malaysian adults, we discovered that 30.6% of respondents scored healthy lifestyle scores of 4–5. We also found that gender, age and ethnicity significantly influenced healthy lifestyle behaviours. To our knowledge, this is the first study in Malaysia to examine the relationship between healthy lifestyle behaviours and socio-demographic factors. Similarly, in a cross-sectional study of a national sample of German adults, Finger et al.^12^ found that the combination of healthy lifestyle that includes daily intake of both fruits and vegetables, adequate physical activity, abstinence from smoking, abstinence from risky drinking and normal body weight increased the overall prevalence of healthy lifestyle with at least four out of five healthy lifestyle factors from 9.3% in 1990–1992 to 14.7% in 2008–2011. Adhering to a healthier lifestyle may have the potential to reduce disease burden and prolong life. However, the percentage of people who have combined healthy lifestyle factors appears to be relatively low globally. Only 10.3% of participants in the US Nurses’ Health Study and the Health Professionals Study had at least four protective lifestyle factors^3^. According to data from the Singapore Chinese Health Study, only 8.5% of the elderly adhered to 4–5 protective lifestyle factors^22^. In our study, about 31% of respondents practiced at least four healthy lifestyle habits. It is possible that a higher prevalence of individuals with healthy lifestyle habits in Malaysia could be explained by specific cultural factors. In general, the prevalence of those with risky alcohol consumption was generally low across the population. The prevalence of alcohol consumption among Malaysian adults is relatively low, which could be attributed to social and cultural reasons. The majority of participants in this study were Muslim (68%) and alcoholic drinks are totally prohibited in Islam. However, in rural Sabah, drinking is important to maintain culture and identity, especially for indigenous communities^24^. Hence, health promotion programs, especially effective alcohol-harm minimization programs, will need to be strengthened to increase population health in those regions. Different strategies are used for different socio-demographic groups, in order to promote ideal lifestyle patterns.

In the current study, we observed a statistically significant association between age and healthy lifestyle behaviours, with younger participants seemingly leading healthier lives than older adults. Similarly, a previous study in Germany also found that younger people had higher overall prevalence of healthy lifestyles than older people^12^. A possible explanation is that increasing age was associated with being physically inactive; also, older people tend to have multiple comorbidities and hence poorer perceptions of their health. The NHMS 2015 reported that half of the Malaysian older population are physically inactive^25^. These studies demonstrate that different intervention strategies may be required for different age groups in the adult population. Another factor contributing to higher healthy lifestyle scores in younger adults may be that middle-aged adults are in the busiest season of life with multiple role demands and responsibilities with regard to both work and family. Middle aged adults are mostly employed with full-time jobs which may leave them with less time to less engage in health-promoting activities, especially physical activity. This showed that middle aged adults do not recognize the need to adopt healthful behaviours and are not health conscious. Previous studies also confirmed that middle aged participants had the most unhealthy lifestyles and higher prevalence of metabolic syndrome^26^. Consequently, we need to pay more attention to middle-aged and older adults, who are at greater risk of developing health problems, in terms of health promotion and disease prevention programs.

The present study revealed unique correlates of healthy lifestyle behaviours for men and women. Females were 3.3 times more likely than males to score 4 to 5 healthy lifestyle factors. Consistent with previous research, gender emerged as a strong predictor of healthy lifestyle scores in the current study. This may be attributed to women having stronger tendencies to be more concerned about health, and are less likely to pick up smoking and drinking habits. For instance, a study conducted in Taiwan showed that women had healthier dietary practices, a higher level of physical activity, lower rates of smoking and obesity, and maintain a healthy metabolic status compared to men^26^. Another possible factor contributing to more prevalent healthy lifestyle scores in women may be that women care more about their diet^27^. Women consumed more fruit and vegetable than men and less rice and sweetened beverages^28^. Moreover, women are more likely to seek professional assistance and more readily accept health advice from medical professionals than men^29^.

There are pronounced ethnicity differences in the healthy lifestyle behaviours. Our findings corroborate with reports that those of Chinese ethnicity were more likely than those of Malay ethnicity to achieve higher healthy lifestyle behaviours. Other ethnicity in Malaysia, in general, were likely than Malays to adopt healthy lifestyle habits. The differences in healthy lifestyle behaviours between ethnic groups may be explained by the complex interaction of socio-economic, cultural and environmental factors^30,31^. In Malaysia, the majority of other ethnic groups live in remote areas where active commuting and farming are the primary mode of transportation and livelihood. These modes of transports and eating behaviours are likely to contribute to higher healthy lifestyle scores in these populations. Previous study suggested that other ethnic groups were more likely than Chinese, Malays and Indians to report being engaged in physical activity. According to a causal model of healthy behaviours among various ethnic groups in Sarawak, Malaysia, the Chinese population were found to possess a better level of healthy literacy, which results in the practice of healthy lifestyle^27^. A systematic review found that Chinese adolescents were associated with higher scores for healthy dietary patterns, which contributed to better diet quality compared to Malays^32^. Thus, ethnic disparities in healthy lifestyle profiles may reflect socio-cultural differences in food preferences, religious beliefs on alcohol consumption, and social influence in physical activity.

Healthy lifestyle behaviours were found to be more prevalent among rural communities compared to urban communities. There is a critical link between communities, the environment, and population health. Studies conducted on knowledge, attitude and practice about non-communicable diseases revealed that rural communities had higher attitude and practice scores as compared to urban people^33^. Rural communities had better disease management strategies including higher willingness to adopt lifestyle changes and access to more robust support networks. On the other hand, urban areas, which can provide more convenient environments and resources such as advanced hospital facilities and skilled healthcare professionals and medical treatments, likely contributed to the dismal situation in which urban residents lack motivation to adopt healthy behaviours. Apart from that, urban environments were also associated with sedentary lifestyles, lack of physical exercise, excess dietary intake of sodium and fat, and increased social drinking as a result of stressful lives^34^. In rural areas, the prevalence of physical activity was higher compared to urban residents^35^. These physical activities are associated with occupations and social activities such as community activities, religious activities, and agriculture. In addition, urban residents consumed fewer servings of fruits and vegetables than rural residents. Easy access to fast food restaurants and higher spending power may predispose urban residents to eat out more often, which translates to higher consumption of foods high in fat and calories, such as meat, processed foods, and fried foods^36^. Therefore, interventions aimed at raising public awareness about the importance of a healthy lifestyle and healthy food consumption could be implemented effectively in urban areas through multisectoral collaboration and participation.

The study has several strengths including national representativeness and a large sample size, thus enabling our findings to be generalised to the adult population in Malaysia. Additionally, participants were interviewed face-to-face by trained interviewers, and the response rate was higher than reported for other studies. However, when interpreting our findings, several limitations should be considered. Except for body weight, all healthy lifestyle factors were self-reported; thus, respondents’ data may be subject to recall bias, resulting in under-reporting of actual behaviour. Furthermore, responses may also be more susceptible to reporting bias, such as an overestimation of desirable health behaviours (e.g. physical activity and fruits and vegetables consumption) and under-report undesirable health behaviours (e.g. smoking and alcohol consumption). Underweight only contributed 5.8% in this study, therefore we did not further analyse as a different category but combined BMI into two categories. Further research should include it as a target sample size to concur this population.

## Conclusion

The small proportion of Malaysian adults with at least four out of five healthy lifestyle habits as reported in this study suggests that additional efforts are needed to encourage Malaysian adults to adopt healthy lifestyles. Each additional healthy lifestyle habit will lead to a further improvement of overall in the general adult population. Understanding how healthy lifestyle behaviours vary by socio-demographic factors such as age group, gender and ethnicity is essential towards developing and implementing effective strategies for increasing the prevalence healthy lifestyles in the community. This study highlights the importance of coordinating actions on health promotion interventions in order to increase the number people with healthy lifestyle behaviours, as well as drive public awareness of how each additional healthy lifestyle factor improves health. Based on findings from this study, there may be merit in considering the development of health promotion campaigns targeted specially at males, older than 30 years, and Malay ethnicity.

## Data Availability

For data protection purposes, the data used for this study are not publicly available but are available from the Institute for Public Health, Ministry of Health Malaysia upon reasonable request and with permission from the Director General of Health Malaysia.
